# Pediatrics

**Published:** 1899-09

**Authors:** Maud J. Frye


### Progress in Medical Science.

### PEDIATRICS.

Reported by MAUD J. FRYE, M. D.

### INGUINAL HERNIA IN CHILDREN.

ECCLES (*British Med. Jour.*, May 13, 1899,) discusses the treatment. Referring to causes he gives as his opinion that cough, constipation, phimosis, and the like, are not the most general and potent factors in producing inguinal hernia, but that improper diet, with the resulting gastro-enteritis, diarrhea, vomiting and over distension of the bowel with gas, is the chief agent in its causation.

In regard to treatment, he considers the radical operation necessary in a small minority of the cases only. It may be advised as follows:

1. Where a child is being properly fed, but in whom a hernia, after a fair trial is not retained by a suitable truss, so that there is little or no chance of a spontaneous cure.

2. Where part of the contents of the sac are irreducible.

3. Where a truss has been worn for at least three years, but with no apparent cure of the protrusion.

4. Where a child has reached the age of at least three years, but has never worn a truss.

5. Where a herniotomy has to be performed for strangulation.

The nonoperative treatment the author believes to be curative in the great majority of cases. He condemns the use of the wool truss, regarding it as inefficient, except in the simplest cases. The spring truss which he advises should be measured for as in the adult, the tape being passed around the pelvis obliquely at the base of the sacrum behind, half way between the crest of the ileum and the top of the great trochanter at the side, and above the symphysis pubis in front. For a single truss the actual number of inches obtained is the correct size; for a double, add one inch more. The adjustment of the truss is of the utmost importance. The whole of the pad of the truss, in order to close the inguinal rings, must lie above the curved line running across the lower abdomen of the child, and known as the fold of Venus. Placed below this it fails to close the canal and pressing the delicate flesh against the pubic bone is liable to cause excoriation. The truss must be worn by the child continually, day and night, sleeping and waking. When removed for cleanliness, the patient must be recumbent and the hernial aperture protected by the nurse's finger or thumb.

Treatment by truss should be begun at the earliest possible moment. It should not be discarded until the child has reached the age of three years. In addition to the use of the truss, attention should be given to the diet, and the bowels carefully regulated. Phimosis may be operated for, if other adequate reasons exist, but its correction will not alone bring about a cure.

---

### THE ANTITOXIN OF DIPHTHERIA AS AN IMMUNISING AGENT.

ADAMS (*Archives of Pediatrics*, June, 1899,) gives the result of the employment of diphtheria antitoxin in 422 cases. Those receiving the treatment were the children admitted as patients to the Children's Hospital in Washington, and the ailments from which they were suffering included almost every variety of surgical or medical disease. The antitoxin was given on account of the repeated occurrence of diphtheria in the institution. The dose given was 100 units to infants, 250 to those between two and six years of age, and from 300 to 500 above this age. Seventeen or 4.28 per cent. of those immunised contracted diphtheria. The maximum duration of immunity was five months, twenty-four days; the minimum, eleven days.

The urine was carefully examined both before and after the administration of the antitoxin. No effect on the kidneys was observed. Urticaria appeared in two cases.

The author believes the dose given for immunising is too small. He feels warranted in asserting that the larger the dose the longer the duration of the immunity.

